# The mediating role of artificial intelligence perception and creative idea generation in the impact of sustainable leadership on digital transformation and circular economy awareness

**DOI:** 10.3389/fpsyg.2026.1892855

**Published:** 2026-10-19

**Authors:** Ceyda Yerdelen-Kaygin, Mustafa Altıntaş, Ethem Topçuoğlu, Onur Oktaysoy, Erdogan Kaygin, Volkan Tatar, Burcu Turan-Torun, Bilal Kobanoğlu

**Affiliations:** 1 Faculty of Economics and Administrative Sciences, Kafkas University, Kars, Türkiye; 2 Faculty of Economics and Administrative Sciences, Kirsehir Ahi Evran University, Kirsehir, Türkiye; 3 Academy of Civil Aviation, Giresun University, Giresun, Türkiye; 4 Faculty of Economics and Administrative Sciences, Istanbul Arel University, Istanbul, Türkiye; 5 Faculty of Tourism, Van Yuzuncu Yil University, Van, Türkiye; 6 Independent Researcher, Giresun, Türkiye

**Keywords:** artificial intelligence perception, circular economy awareness, creative idea generation, digital transformation, sustainable leadership

## Abstract

**Introduction:**

Today, sustainable leadership is a strategic leadership approach that addresses environmental, social, and economic priorities from a holistic perspective while at the same time driving digital transformation processes. The research aims to explain how sustainable leaders mobilize organizational resources, technological infrastructure, and human creativity within the framework of Dynamic Capabilities Theory.

**Methods:**

The research was conducted on 456 white-collar employees working in small and medium-sized enterprises operating in Giresun, Kars, and Kırşehir provinces. The data collected by the convenience sampling method were analyzed using the PLS-SEM method with the SmartPLS 4.1.1 program.

**Results:**

The analysis results show that sustainable leadership has significant and positive effects on perceptions of artificial intelligence, the generation of creative ideas, digital transformation, and awareness of the circular economy. It was found that the perception of artificial intelligence (AI) has a significant effect on both digital transformation and awareness of the circular economy. It was also determined that creative idea generation has significant effects on digital transformation and awareness of the circular economy. The results of the mediation analyses revealed a partial mediating role for all variables.

**Discussion:**

The findings reveal that sustainable leadership is not limited to the environmental and social responsibility dimension and is a strategic element that directs digital transformation processes, shapes perceptions towards artificial intelligence, and encourages organizational creativity.

## 1 Introduction

The 21st century is a time of rapid transformation in the business world and social structure. Assuming that one of the basic building blocks of this transformation is digitalization, it can be said that the other is sustainability and economic structure. The effects of the climate crisis, the rapid consumption of resources, the increase in environmental sensitivity, and the expectations in society make it imperative for organizations to develop strategies in line with sustainability (Geissdoerfer et al., 2020). It has been determined that weather-related extremes in the European Union region due to climate change caused 822 billion Euros of damage between 1980 and 2024. Considering that approximately 25% of this loss occurred between 2020 and 2024, it is seen that economic losses due to climate change are increasing day by day (European Environment Agency (EEA), 2025).

The increase in the loss of life, along with material losses, has made environmental transformation processes mandatory. Since the transformation processes are quite comprehensive, leadership understandings are also changing, and the need for leaders who use technology effectively, respond quickly to social expectations, and have high environmental sensitivity has increased, instead of traditional leadership understandings (Liao, 2022). As one of these leadership styles, sustainable leadership is a leadership approach that encompasses social and economic sustainability as well as environmental sustainability. Along with leadership, new production and consumption models that prioritize creativity and efficiency in organizations have become widespread (Ahsan and Khawaja, 2024). One of these models is the circular economy model, which, unlike a linear structure, is based on waste and resource efficiency in production and consumption, and offers a sustainable and innovative approach. Circular economy awareness involves organizations developing innovative business models and creating economic added value (Castro-Lopez et al., 2024). It is seen that large investments are made in digital transformation and AI in order to spread new technologies that support sustainability in the organization. AI is one of the most influential variables today and is a technological tool that is used effectively in many areas, from decision-making processes to the suitability of production, from customer analysis to human resources functions for organizations. Applying, managing, and sustaining AI in an appropriate way for employees in organizations is considered important in every aspect (Huang et al., 2019). In addition to leadership, technological developments, and many other factors, employees’ creative ideas are seen as one of the competitive factors in today’s world. Creative idea generation has taken its place as an important element in an increasingly digitalized business world, trying to cope with environmental concerns (Amabile and Pratt, 2016).

The primary objective of this study is to examine the impact of sustainable leadership on digital transformation and awareness of the circular economy, and to identify the potential mediating roles of perceptions of AI and creative idea generation in this impact. Although there are a few studies in the literature that evaluate these variables individually or in pairs, there is still a lack of research explaining the impact of sustainable leadership on new technological and social developments such as the circular economy, digital transformation, and artificial intelligence. While addressing variables piecemeal yields limited results, considering multiple variables simultaneously allows for a more thorough explanation of how these variables function by leveraging statistical differences. In this study, the concepts of digital transformation, the circular economy, perceptions of artificial intelligence, and creative idea generation, which currently represent both opportunities and threats for organizations, are evaluated through the lens of sustainable leadership. This study, which seeks to explain the functioning of these variables in a holistic manner, draws upon the Dynamic Capabilities Theory. This theory is a significant framework for examining organizations’ ability to adapt to rapidly changing market and environmental conditions. One of the unique aspects of this study is its holistic approach to current issues in the field of organizational behavior. For example, it is well known that governments are using carbon taxes to compel organizations to adopt circular economic practices. Organizations are affected by market conditions, while government policies such as Society 5.0, Industry 5.0, and “Made in China” are also compelling organizations to undergo a digital transformation. These imperatives represent a struggle for survival for organizations. This raises the question of what organizations must do. The expectation that AI will find solutions or facilitate solutions to current challenges is frequently discussed in the literature. While this study supports the existing literature, it also demonstrates that human-centered creative idea generation remains a more significant behavioral pattern compared to artificial intelligence. Furthermore, the research findings support the idea that the concept of leadership is redesigning itself in line with environmental and technological transformations, and that dynamic skills are now a leadership necessity.

## 2 Literature review

### 2.1 Sustainable leadership

Sustainable leadership is a leadership style that has emerged as a response to many changing, challenging conditions such as changing business life, globalization, complexity, technological developments, performance pressure, etc. (Rosário and Boechat, 2025). The reason why sustainable leadership emerged in response to these challenges is that it aims to provide innovative and sustainable solutions by improving the perspectives, awareness, thinking, and perception of leadership and followers (Waqar et al., 2023). Sustainable leadership has a structure that inspires followers, defines the working environment, and aligns the needs of the followers and the organization (Gjerde and Ladegård, 2019). In addition, sustainable leadership plays an important role in promoting innovation (Iqbal et al., 2022). The point of consensus in the definitions of sustainable leadership is sustainability, and many people have rallied around the idea of further implementation of sustainability. In this respect, it is stated that an effective planning and management approach is needed to develop human resources, provide quick access to resources, train employees, and make many elements suitable for sustainability (Khan et al., 2023).

Sustainable leadership is related to the ability to respond to where organizations are in terms of sustainability, and there are expectations that sustainable leaders act in an environmentally and socially responsible manner (Avery and Bergsteiner, 2011; Narayanan et al., 2021). In essence, sustainable leadership is a leadership style that partially carries the characteristics of transformational leadership, but also includes elements such as intellectual development of employees and adoption of innovative approaches (Christensen et al., 2013). On the other hand, sustainable leadership, which has similar aspects to the responsible leadership style, is claimed to help subordinates with sustainability, innovative solutions, and knowledge sharing (Ramus and Steger, 2000; Doh and Quigley, 2014). Sustainable leadership has an approach that focuses on creating long-term value for the organization by balancing the goals of society, economy, and environment (Liao, 2022). When the research on this leadership style is examined, it is possible to say that organizations based on sustainable leadership principles create sustainable performance outcomes and resilience in the long term. Sustainable leadership is a leadership style that is under intense pressure from external stakeholders rather than internal stakeholders. This is because sustainable leadership is seen as a bridge between an organization’s economic goals, environmental protection, and social justice (Raj et al., 2020).

### 2.2 Digital transformation

Although the foundations of digital transformation date back to the widespread use of computers, it actually became famous with the emergence of Industry 4.0. Although Industry 4.0 was introduced as a new national policy in Germany, it has made an impact in the world as a roadmap foreseeing the basic needs of 2020 (Kim et al., 2021). In today’s digitalized world, traditional practices and paradigms have been and continue to be replaced by digital tools. Digital transformation is not seen as a situation or just an option for organizations to pursue, but as a strategic necessity to gain competitive advantage, to survive, and to be successful (Van Veldhoven and Vanthienen, 2023). The main reason for the dizzying pace of change and transformation of modern business life is the relentless progress of digital innovations (Owoseni, 2023). This progress is not as easy as the transition from a traditional organizational structure to digital transformation. It requires an understanding of the complex interactions between elements such as leadership, technology, culture, and strategy in organizations (Omol et al., 2025). In addition, digital transformation is not only about adopting technology but also about adapting the working principles of organizations and customer interactions to the digital world (Pricopoaia et al., 2025).

Digital transformation is defined as the process of building capabilities to enable continuous and strategic renewal of organizations (Warner and Wäger, 2019). Digital transformation encompasses digitalization and numerical data and leverages human capital, stakeholders, and data-driven decision-making processes to achieve set goals (Gong and Ribiere, 2021). It is reported that organizations may face some challenges, such as ensuring that the digital transformation they undertake aligns with their organizational strategies, that digital skills can be developed, and that they are compatible with international standards in order to benefit from the benefits and distribute the risks brought by digital transformation (Goodwin, 2018). Especially for employees, it is suggested that the adoption process of digital competencies and technologies should be maintained in a healthy way; psychological problems may occur (Harish et al., 2023).

### 2.3 Circular economy awareness

The economic models adopted by states in the process from the Industrial Revolution to the present day are referred to as the linear economy. The linear economy includes an economic model in which resources can be taken and transformed into products in an unlimited way, consumed, and discarded. The circular economy model, on the other hand, has a structure that aims to close the flow of products and energy within the scope of the economy (Delchet-Cochet, 2020). In particular, the circular economy benefits from the sustainability put forward by society and the business world, while the linear economy is argued to be losing this feature in social, financial, and ecological terms (Weetman, 2020).

The concept of circular economy first emerged towards the end of the 20th century as an alternative structure to the traditional economic model and was first overshadowed by the People’s Republic of China until the “Circular Economy Promotion Law” was enacted (Lieder and Rashid, 2016). After this law was enacted by China, the circular economy concept attracted the attention of companies around the world, and with the realization of its potential, they started to consider the idea that it could be an alternative to the existing economy (Liakos et al., 2019). The main purpose of the circular economy is to eliminate waste by creating a network of closed-loop systems that address processes such as the production, use, repair, and reuse of products in a cycle. In doing so, it aims to minimize recycling, although the idea of complete elimination cannot always be implemented. In this respect, it contrasts with the linear economy (Scarpellini et al., 2020).

In the context of the circular economy, individual actions play an important role in shaping the strategies developed to achieve the goals of the circular economy (Appolloni et al., 2022). Therefore, it is important to understand the awareness levels of individuals in terms of the circular economy. Individuals are the actors of change (Abeysekera, 2022). In addition to individuals, the adoption of circular economy understanding from the beginning is a structure in which many stakeholders, from organizations to policymakers, from academics to society, act together and partner in the transformation process (Nowicki et al., 2023). Therefore, instead of addressing circular economy awareness from a single point, it is necessary to look at the issue holistically and give importance to comprehensive studies. In addition, it is not possible to ignore the awareness of the individual in terms of circular economy strategies (De Fano et al., 2022).

Looking at the research in the literature, for example, it is stated that consumers in Europe perform less sustainable purchasing behavior due to their low level of awareness of circular strategies (Otto et al., 2021). On the other hand, in a study conducted in China, it is argued that the interactions are not understood due to the lack of effective guidance during the transition from a linear economy to a circular economy (Lanau and Liu, 2020). In this respect, the development of the circular economy at the global level is directly affected by individuals’ preferences, lifestyles, perceptions, and awareness (Almulhim and Abubakar, 2021). In terms of awareness, there are studies in the literature on circular economy and sustainability (Szilagyi et al., 2022). It is argued that the main obstacles to the circular economy are a lack of awareness and knowledge, and it is stated that the fact that the circular economy is accepted and applicable in the world is related to individual awareness (Lahane et al., 2021).

### 2.4 AI perception

AI has been widely used and accepted over the last decade (Alvarez et al., 2024). A report by the International Data Corporation (IDC) predicts that spending on AI will reach approximately 100 billion dollars in 2023. Similarly, a recent forecast by the IDC shows that the worldwide AI software market will grow from $64 billion in 2022 to approximately $251 billion in 2027. This forecast was made as of the end of 2023 (He, 2025). Finally, in its report published in October 2024, it is predicted that global spending on AI-enabled technologies will reach 337 billion dollars. In addition, the importance of the trend towards AI is emphasized in this report (IDC, 2004). Statistics from international organizations show that AI is being adopted on a large scale across the globe, and its rapid diffusion is having a significant impact on society. In this respect, individuals are increasingly adopting and trusting the recommendations and decisions made by artificial intelligence. These recommendations can be as simple as a friend or a movie recommendation, or as complex as the use of autonomous vehicles for transportation (Minh et al., 2021).

In the past, AI was generally used to describe machines or computer systems that can exhibit human-like intelligence (Tchuente et al., 2024). Today, with the emergence of modern data-driven approaches (machine learning, deep learning, reinforcement learning, etc.), AI has become an inevitable element in all segments of society (Kamm et al., 2023). Similar to the concept of artificial intelligence, the term “artificial creativity” has been proposed, as it involves machine learning and machine-based output (Runco, 2023). In addition, the characteristics of artificial creativity have been examined in academic research, and the characteristics of AI have been compared with human creativity (Cropley et al., 2022).

AI technology has become an important field, especially with ChatGPT at the end of 2022. Because of this technology, creativity has evolved into a different dimension, and it has started to be examined whether the ideas produced by AI are original or not. Many factors, such as comparing these ideas with the ideas produced by humans and shaping the facts put forward by AI and humans according to the perception of other people, have come to the fore (Koivisto and Grassini, 2023).

In addition to artificial intelligence, the concept of productive AI is expressed as a driving force for the adoption of AI at the corporate level (Kshetri et al., 2024). It is stated that recent developments in the field of AI are mainly the result of the emergence of Productive AI (Sengar et al., 2025). In general terms, generative AI refers to an AI system that can create new content with text, visual tools, or other media tools (Susarla et al., 2023). The important point here in productive AI is that it is handled at the institutional level and has a structure that provides a completely different innovation across all functions of organizations (Dwivedi et al., 2024). It is important that productive AI is usable and sustainable. Especially in the usability of artificial intelligence, AI literacy and education are of great importance. It is also stated that it is critical for both individuals and organizations to use AI effectively (Howard and Gugger, 2020).

### 2.5 Creative idea generation

Creativity is a concept that refers to the ability to promote change and the ability to overcome change. Creativity is the capacity to generate an idea that fits both the essence of the work and its context (Jung and Vartanian, 2018). Creativity generally refers to the ability to bring it out; that is, the basic abilities related to language use, and it is suggested that creativity may be related to this (Beaty and Kenett, 2023). While the concept of creativity involves the production of original and useful works (Runco and Jaeger, 2012), idea generation is the basis of creative thinking that reveals creativity (Sowden et al., 2015).

Although individuals have the capacity to think creatively, they can often remain passive in terms of idea generation (George and Lasher, 2024). When individuals try to think creatively or generate creative ideas, their initial tendency is based on ideas and approaches that are easily accessible in their minds and that they usually use (Storm et al., 2020). It is stated that creative idea generation is a top-down process that requires the participation of executive functions. It is also reported that this is handled within the controlled attention theory of creative cognition (Beaty et al., 2014). Although creative idea generation involves complex cognitive processes (Benedek and Fink, 2019), it is explained that individuals continuously extract task-related information from their long-term memory and keep it in their mental working memory at the point of generating new ideas (Lu et al., 2022).

## 3 Theoretical background and hypothesis development

With the rapid development of technology, there has been a great change in organizations, but the emergence of the global climate crisis and other social problems due to people’s consumption frenzy has created other situations that need to be solved within the organization (Kinzig et al., 2013). Reducing CO_2_ emissions, carbon tax, more environmentally friendly production, fulfillment of obligations to meet ISO 14001 environmental management standards, obligation to use solar energy panels for buildings, and many other new practices are challenging for SMEs. In the current organizational environment, where there are many negative as well as positive factors for SMEs, dynamic capabilities come to the forefront (Ikram and Sadki, 2024). Dynamic capabilities theory (DCT) suggests that organizations restructure their resources in order to perceive changes in their environment, seize opportunities, and achieve sustainable competitive advantage (Teece et al., 1997).

Dynamic capabilities refer to the simultaneous and sustainable tracking of an organization’s economic, environmental, technological, and social capabilities and the modification and enhancement of capabilities in response to change (Mishra and Kiran, 2025). DCT provides a framework for how to use digital transformation, artificial intelligence, circular economy, and creative idea generation to capture, adapt, integrate, and restructure opportunities to ensure the organization’s competitive advantage, competence, effectiveness, and sustainability (Malakar et al., 2025). The theory of dynamic capabilities examines how rare, inimitable, and irreplaceable capabilities should be systematically sustained, integrated, developed, and restructured in rapidly changing market conditions. As technologies, customer preferences, forms of competition, and regulatory frameworks change, capabilities are expected to adapt accordingly. From this perspective, the process, position, and pathways are considered to form three interconnected dimensions. Processes refer to the structure within which organizational activities are carried out, coordinated, learned, and transformed. Position describes the organization’s standing in the market in terms of technology, intellectual capital, complementary resources, finances, and reputation. Paths, on the other hand, refer to the entire set of methods the organization has followed from its past to the present. These methods can be categorized as prior investments, business relationships, accumulated knowledge, and organizational routines (Teece et al., 1997).

### 3.1 Relationship between concepts

Especially in terms of sustainability, it is seen that sustainability is closely related to many concepts such as digital entrepreneurship, circular economy, smart production, artificial intelligence, and digital technologies, which are closely related to the concept of digital transformation (Ajwani-Ramchandani et al., 2021). In addition, it is possible to say that many elements, such as innovation, R&D, and sustainability in SMEs, have taken their place in the literature (Isensee et al., 2020). In this respect, it can be said that the ability to use digital capabilities is the basis of the socio-economic and environmentally oriented approach to sustainability. In addition, it is known worldwide that digital transformation is not only about technology, but also plays an important role in the realization of industrial paradigms and in the contribution of organizations to the social, environmental, and circular economy (Payer et al., 2024).

AI applications are thought to help create a more sustainable world by creating a balance between production and consumption. AI technologies provide significant advantages in terms of speed, efficiency, and data analytics in today’s business world. However, it can also be said that it has led to a transformation in the understanding of leadership. In terms of leadership, it is reported that they should evaluate AI applications not only as an automation tool but also as an innovation within the framework of ethical principles (George et al., 2021). When the literature is examined, it is seen that there is a relationship of 0.46 between sustainable leadership behavior and AI ethics (Aldighrir, 2024). A relationship of 0.62 was determined in the evaluation that the use of AI will have a significant contribution to the development of sustainable leadership in health organizations (Movahed and Bilderback, 2024). However, it is seen that the works mentioned in the literature are not exactly equivalent to the subject examined, and there is no work in the literature that meets the expectations and fully explains the relationship between the variables. Dynamic Capabilities Theory is used in the study to explain the relationship. Based on the theory, it is thought that AI provides a great transformation in the organizational environment, and the organization responds by adapting and integrating its resources, capabilities, and functions to the new situation (Gao et al., 2025). Although transformation is a challenging process for the organization, it is expected that a sustainable leader who overcomes it will be effective in the organization. Based on this, hypothesis H1 was formed.

H1: Sustainable leadership has a significant effect on the perception of artificial intelligence.

The circular economy contributes to the organization in terms of reducing the need for raw materials and reducing costs through recycling, remanufacturing, and waste reuse. While the circular economy creates a significant opportunity for the organization in terms of resource efficiency and cost savings, an unplanned approach may result in quality degradation and customer loss. For this reason, managing the circular economy-oriented transformation will be possible with a leadership approach compatible with green transformation (Bashynska et al., 2024). Sustainable leaders are expected to bring together employees, organizations, government, and international organizations to create an environmentally friendly and green organization. By contributing to the reorganization of the supply structure of the organizations by the leaders, the circular economy understanding becomes widespread within the organization (de Andrade Júnior et al., 2020). Due to the limited literature and lack of empirical findings, the interaction between the variables is theoretically based. One of the current aspects of the theory of dynamic capabilities is the evaluation of opportunities. AI creates an opportunity for organizations, and the organization mobilizes its resources to capitalize on that opportunity. Leadership and managerial values come to the forefront in order to perceive opportunities and understand transformation. Within the scope of Dynamic Capabilities Theory, it is expected that recycling and reuse issues are compatible with the dynamic orientation of resources, and sustainable leaders are expected to be the factor that will affect this effect positively or negatively (Alka et al., 2025). Based on the existing theoretical basis, hypothesis H2 was formed.

H2: Sustainable leadership has a significant impact on circular economy awareness.

Digital transformation is a multifaceted process that brings together organizational culture, strategic innovation, and technology. Organizations that adopt sustainability practices and are led by leaders with advanced digital skills transform their business models and sustainability approach by achieving higher operational efficiency and competitiveness (Sacavém et al., 2025). From this point of view, it is thought that a sustainable digital transformation will be created under the leadership of the leader by activating the dynamic capabilities within the organization. Although this idea has been put forward, there are, of course, different realities of life. The annual amount spent by organizations on digital transformation is expected to be around 3.9 trillion dollars in 2027. The annual loss suffered by organizations as a result of cyber-attacks is around 8.15 trillion dollars (Catal et al., 2025). In addition, considering that the success rate of digital transformation is around 30%, the need for a leader in the realization of digital transformation is emphasized (Yerdelen-Kaygin et al., 2025). For dynamic capabilities to emerge effectively within organizations, the accumulation of experience, the occurrence of organizational learning, the exercise of managerial initiative, the soundness of managerial decisions, and the sharing of information take center stage. Leaders are one of the most important factors in determining which opportunities to pursue, how to allocate resources, and when to transform existing organizational structures. H3 was established because it is expected that a successful digital transformation process will be carried out by bringing the dynamic capabilities of organizations to the forefront.

H3: Sustainable leadership has a significant impact on digital transformation.

Sustainable competitive advantage constitutes one of the important strategies for organizations to reach better positions in the market or to maintain their current position. In order to create a sustainable competitive advantage, it is necessary to produce innovative services and products in accordance with customer demands within the organization (Javed et al., 2021). The sustainable leader is expected to create a suitable environment for the production of creative ideas within the organization, with the effectiveness of the positive ties created socially and environmentally (Iqbal and Piwowar-Sulej, 2022). Because there are many failures and serious financial investments during the creation of a viable creative idea, there is a challenging process that requires the leader to constantly motivate and support many people in generating creative ideas and to convince stakeholders in this regard. Javed et al. (2021) found a moderate interaction of 0.41 between sustainable leadership and creative self-efficacy. Carmeli et al. (2013) also found a moderate interaction of 0.44 between leadership and creative problem solving. Previous studies reveal the power of sustainable leaders to mobilize the organization and their ability to direct dynamic capabilities, and the H4 hypothesis was formed based on this.

H4: Sustainable leadership has a significant effect on creative idea generation.

Despite its data-driven, complex nature, AI generates valuable insights for organizations and opens up many opportunities, including in competitive, time-sensitive markets with low profit margins (Ajmera et al., 2025). AI technology has opened the door to a major shift in addressing environmental and economic challenges. Thanks to machine learning and real-time data analysis, it is seen in Industry 5.0 that AI is effective in the formation of more efficient and effective systems, with minimum waste in the production and supply chain (Hernandez et al., 2025). Although AI has great potential in terms of creating and managing circular economy awareness, it is thought that an AI policy that is not designed specifically for the organization will create danger for the organization. Because AI technology still has hallucination problems (Sun et al., 2024). If AI is not designed according to the organization’s specific characteristics, it will merely constitute a run-of-the-mill capability, as it will not create any advantages beyond the more efficient management of existing tasks. For AI to create a dynamic capability, it must transform the organization’s resource base and the way it conducts business. At the same time, creating and shaping AI as a dynamic capability will help resolve current issues related to it, such as hallucination. Although there are some problems, the fact that AI is a dynamic capability for the organization continues, and since it is expected to contribute to the circular economy, hypothesis H5 has been formed.

H5: AI Perception has a significant effect on circular economy awareness.

The perceptions of leaders and employees towards AI technologies create both convenience and negativity in adapting new technologies to the organization (Ahn and Chen, 2022). It is known that many effects, such as technostress, AI anxiety, fear of unemployment, and burnout, affect employees due to artificial intelligence. AI is a candidate to become an indispensable part of organizations by transforming the basic business processes of every sector in the next few years (Okunlaya et al., 2022). Considering the studies conducted in today’s conditions, it can be said that a medium-level interaction between AI and digital transformation is predominant (Abou Hashish and Alnajjar, 2024). Considering the report prepared by the United Nations, it is seen that the concept of digital transformation is gradually mentioned together with artificial intelligence. According to the report, the AI market, which was 2.5 trillion dollars in 2023, is expected to reach 16.4 trillion dollars in 2033. At the same time, 40% of the jobs undertaken by humans are expected to be fulfilled by AI (United Nations Conference on Trade and Development, 2025). Based on this, hypothesis H6 has been formed.

H6: AI Perception has a significant effect on digital transformation.

The circular economy is a concept associated with productivity and performance, which is the result of the ecological, economic, and organizational contributions of new technologies and practices (Sierra-Pérez et al., 2021). Despite the growing interest in the circular economy, the global economy continues to become less circular. This contradiction is said to be due to the neglect of the creativity and innovation relevance of circular economy management strategies (Mignacca et al., 2025). The theory of dynamic capabilities also supports this view to a certain extent. Within the framework of dynamic capabilities, organizational processes such as product development, strategic decision-making, and resource restructuring take shape in a simpler, experience-based, and more flexible manner in rapidly changing environments; as a result, these processes may take longer to complete. As this statement also indicates, creativity and innovation are the main drivers of the circular economy, but organizational factors can delay their implementation. H7 hypothesis is formed with the generation of creative ideas as a dynamic resource on the circular economy.

H7: Creative idea generation has a significant impact on circular economy awareness.

Creative idea generation is an important driver of organizational success and represents an effective cognitive structure for the diffusion of innovation from the individual to the entire organization. Creative idea generation, in essence, describes the conceptualization of something that has never existed before or the re-expression of an existing idea with different combinations, with the use of imagination (Lazzeretti et al., 2025). Digital transformation is an important factor in bringing dynamic capabilities to the organization that facilitates creative idea generation (Mele et al., 2024). Based on this, hypothesis H8 was formed.

H8: Creative idea generation has a significant impact on digital transformation.

As can be seen from the accumulation of knowledge in the literature, environmental and technological sustainability are among the key elements of organizational success in today’s business world, and in this context, sustainable leadership drives digital transformation and circular economy awareness with the principles of long-term value creation, ethical management, and stakeholder orientation. The effectiveness of sustainable leadership is closely related to employees’ perceptions of technological developments and the support of creative ideas in the organization. Sustainable leaders emphasize that digital transformation is a process of cultural change by evaluating technologies such as AI and big data not only in technical but also in ethical and human dimensions. Based on this information, the research hypotheses are given below.

H9: AI Perception has a significant mediating role in the effect of sustainable leadership on circular economy awareness.

H10: AI Perception has a significant mediating role in the effect of sustainable leadership on digital transformation.

H11: Creative idea generation has a significant mediating role in the effect of sustainable leadership on circular economy awareness.

H12: Creative idea generation has a significant mediating role in the effect of sustainable leadership on digital transformation.

In accordance with the hypotheses, the research model is presented in Figure 1.

**Figure 1:**
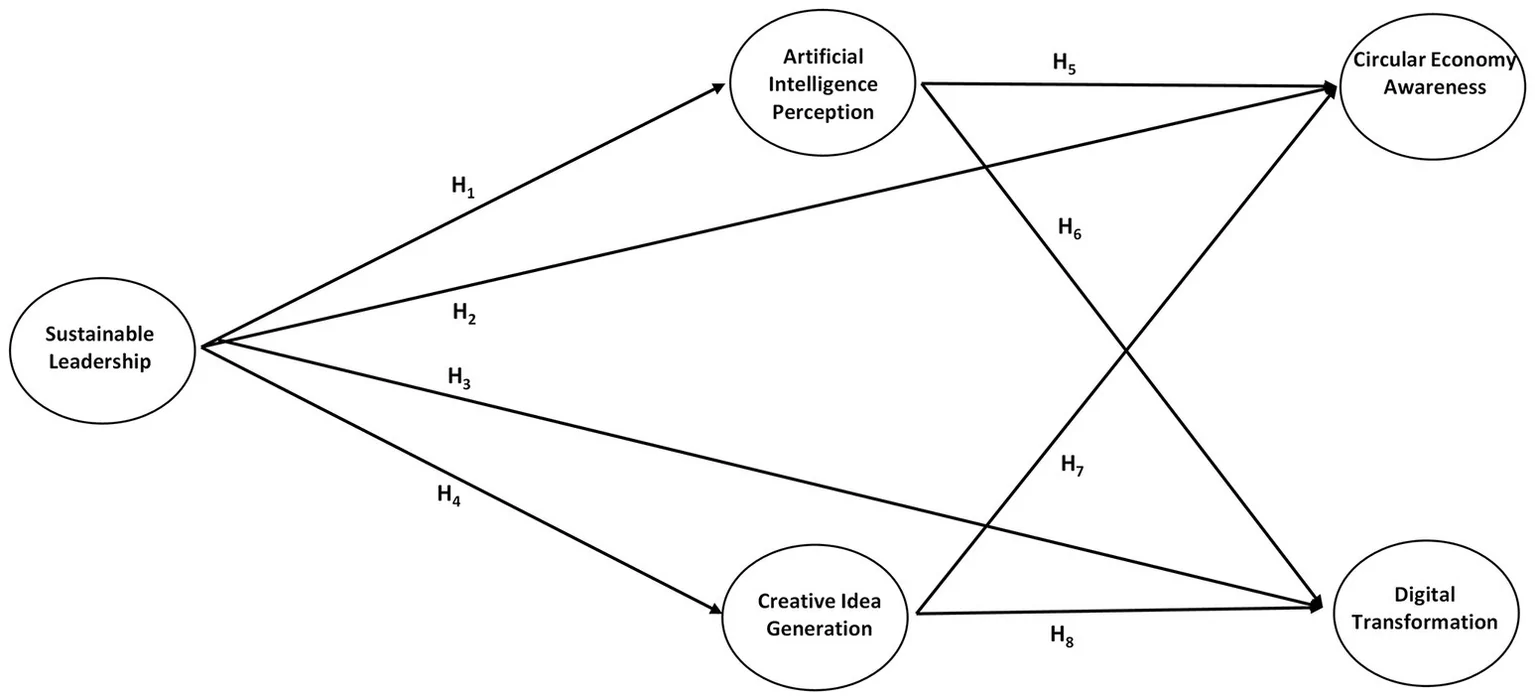
Research Model.

## 4 Method

The population of this study consists of white-collar employees of small and medium-sized enterprises operating in Giresun, Kars, and Kırşehir provinces. According to the Turkish Statistical Institute, SMEs alone account for 68.5% of personnel employment in Turkiye (Turkstat, 2024). This situation of SMEs is similar to that of other countries in the world. In this study conducted on SMEs, the survey technique was applied with convenience sampling. For the research, permission was obtained from the Giresun University Social Sciences, Science and Engineering Sciences Research Ethics Committee with the Board Decision dated May 09, 2025, and numbered 05/160. The permission decision was documented with the letter of Giresun University dated May 09, 2025, and numbered E-50288587 − 050.01.04-86274. Ethics committee permission was obtained before the start of the research, and the survey was started after the Board Decision was obtained. All processes were carried out in accordance with the Helsinki Declaration criteria. All information from the participants was collected anonymously. In all scales used in data collection, a 5-point Likert scale ranging from 1 = Strongly Disagree to 5 = Strongly Agree was used. The data obtained as a result of the survey were analyzed with the PLS-SEM model using the SmartPLS program version 4.1.1. SmartPLS program was preferred due to its suitability for evaluating models with a large number of components and indicators (Hair et al., 2022).

### 4.1 Sample selection

Data were collected from 43 different SMEs in Giresun, 14 different SMEs in Kars, and 52 different SMEs in Kırşehir between June 02, 2025, and December 10, 2025. In order to determine the minimum number of participants to represent the sample, which is thought to represent approximately 250,000 employees, the following Equation 1 was used by Cochran (1977), taking into account the 95% confidence level and 5% margin of error.

$$n_{0}=\frac{Z^{2}\mathrm{pq}}{e^{2}}\;\;$$ (1)

In this context, according to the calculation made using the formula, at least 384 people should be reached. SMEs were visited first, and printed survey forms were distributed to those that agreed to participate in the study. Two weeks after the initial forms were distributed, the documents were collected; however, since the number of completed surveys was insufficient, the SMEs in question were visited repeatedly to ensure that those who had not previously participated in the survey did so. The survey and consent forms were numbered to prevent duplicate submissions. Nevertheless, 24 survey forms were invalidated after it was determined that they had been filled out more than once based on the consent forms. In the study, 456 white-collar employees were reached by using a convenience sampling method.

### 4.2 Measurement instruments

The Sustainable Leadership Scale was developed by Achmad and Inrawan Wiratmadja (2024). To adapt it into Turkish, the terms were first translated into Turkish and then back-translated into English to verify their meaning. In addition to the authors, other individuals in Turkey also adapted the scale into Turkish during the same period (Şahin and Esenkaya, 2026). The scale consists of six statements and one dimension. The Cronbach’s alpha value of the original scale was determined to be 0.861. The scale includes statements such as “Leaders in our organization act in a socially responsible manner.”

The AI Perception Scale, developed by Kurtboğan and Ak (2024), was used to measure the perception of artificial intelligence, and the scale consists of ten statements and one dimension. Since the original was in Turkish, it was not translated. The Cronbach’s alpha value of the original scale is 0.931, and the scale includes statements such as “I believe that artificial intelligence will reduce the workload of employees.”

The Creative Idea Generation Scale developed by Prajogo and Ahmed (2006) was used to measure creative idea generation. To adapt it into Turkish, the terms were first translated into Turkish and then back-translated into English to verify their meaning. The scale consists of four statements and one dimension. The Cronbach’s alpha value of the original scale was determined to be 0.820. The scale includes statements such as “In our company, employees frequently encounter non-routine and challenging work that stimulates creativity.”

The Circular Economy Awareness Scale developed by Gonella et al. (2023) was used to measure circular economy awareness. To adapt it into Turkish, the terms were first translated into Turkish and then back-translated into English to verify their meaning. The scale consists of eight statements and two dimensions. The Cronbach’s alpha value of the original scale was determined to be 0.870. The scale includes statements such as “I am aware of the benefits of using remanufactured products for Circular Economy Awareness principles (e.g., Amazon, which sells remanufactured printer toners).

The Digital Transformation Scale developed by Yao et al. (2024) was used to measure digital transformation. The scale was previously translated into Turkish by Yerdelen-Kaygin et al. (2025). The scale consists of four statements and one dimension. The Cronbach’s alpha value of the original scale was determined to be 0.917. The scale includes statements such as “In the past three years, our company has significantly improved the existing production and operation processes by using digital technologies such as big data, cloud computing, the Internet of Things, artificial intelligence, etc.”

### 4.3 Generalizability of the sample

In recent studies, some analyses have been used to show the generalizability of the sample. Although this study covers three provinces and many enterprises, it is thought that it may have some limitations in terms of generalizability (Liengaard et al., 2021). Cross-validation predictive ability test (CVPAT) with PLS-SEM is used as an indicator for measuring generalizability capacity. In this context, two models are proposed; the first model uses an assessment based on the Indicator Average to ensure a minimum prediction accuracy, called naive analysis. The second model is based on the Linear Model, which provides a more robust validation (Capeau et al., 2024). In this context, information on the analysis for generalizability based on the indicator average is presented in Table 1.

**Table 1:** Indicator average analyses.

Items	PLS loss	IA loss	Average loss difference	t value	*p* value
AI Perception	0.805	0.977	−0.172	5.953	0.000
Creative Idea Generation	0.676	0.967	−0.291	7.069	0.000
Circular Economy Awareness	0.757	1.025	−0.267	6.557	0.000
Digital Transformation	0.580	1.026	−0.446	8.390	0.000
Overall	0.730	0.999	−0.269	9.404	0.000

The fact that the *p*-values in Table 1 are less than 0.05 and t-values are greater than 1.96 at the 95% significance level indicates that the sample meets the minimum requirements for external validity and generalization (Hair et al., 2022). The analysis of the linear model is presented in Table 2.

**Table 2:** Linear model analyses.

Items	PLS Loss	LM loss	Average loss difference	t value	*p* value
AI Perception	0.805	0.816	−0.011	1.664	0.097
Creative Idea Generation	0.676	0.681	−0.005	0.582	0.561
Circular Economy Awareness	0.757	0.775	−0.018	2.807	0.005
Digital Transformation	0.580	0.597	−0.017	2.219	0.027
Overall	0.730	0.743	−0.013	3.086	0.002

When Table 2 is examined, it is seen that the AI Perception and Creative Idea Generation variables do not meet the adequacy for external validity since their *p*-values are greater than 0.05. It was determined that the model provided strong external validity with the Circular Economy Awareness and Digital Transformation scales. The results obtained show that the research model statistically meets the naive and strong generalizability limits.

### 4.4 Common method bias and multi-correlation

Common method bias refers to the negative impact of systematic measurement errors arising from the method on the findings in quantitative analyses. Common method bias is related to bias in the validity, reliability, and parameter estimates of variables with other variables. In this context, common method bias has detrimental effects on findings by inflating or deflating correlations between variables. In order to measure common method bias, Harman’s one-factor analysis was conducted through the SPSS program. In this context, the number of factors was selected as one with the principal axis factoring method, and a result of 41.601% was obtained from the analysis. In the analysis, the fact that all of the variables had an explanation rate below 50% under a single factor indicated that there was no common method bias (Hair et al., 2019). In addition, PLS confirmatory factor analysis was conducted through the SmartPLS program for the detection of common method bias, and the Variance Inflation Factor (VIF) values of the variables were obtained (Hair et al., 2022). Values related to the analysis are presented in Table 3.

**Table 3:** Common method bias analysis.

Items	VIF
AI Perception → Circular Economy Awareness	1,664
AI Perception → Digital Transformation	1,664
Creative Idea Generation → Circular Economy Awareness	2,080
Creative Idea Generation → Digital Transformation	2,080
Sustainable Leadership → AI Perception	1,000
Sustainable Leadership → Circular Economy Awareness	2,260
Sustainable Leadership → Creative Idea Generation	1,000
Sustainable Leadership → Digital Transformation	2,260

Since the VIF values between the variables are below 3, it is determined that there is no common method bias. In addition to common method bias, the effect of multiple correlations should also be examined. The values related to the correlation analysis performed with the SPSS program are presented in Table 4.

**Table 4:** Correlation analysis.

Items	1	2	3	4	5
AI perception	1				
Creative idea generation	0.545^**^	1			
Circular economy awareness	0.606^**^	0.709^**^	1		
Digital transformation	0.585^**^	0.690^**^	0.729^**^	1	
Sustainable leadership	0.593^**^	0.696^**^	0.662^**^	0.786^**^	1

Since there is no value above 0.90 in the values presented in Table 4, it is determined that there is no multicollinearity problem (Hair et al., 2019).

## 5 Findings

In the study, a total of 456 white-collar employees were reached, and detailed socio-demographic information of the participants is presented in Table 5. The majority of the participants were male (77.60%), married (66.20%), and between the ages of 31 and 40. It is seen that these values are in line with the current business structure in Turkiye, as women are less involved in business life.

**Table 5:** Demographic variables.

Demographic	Variable	n	%
Gender	Female	102	22.40
	Male	354	77.60
Marital status	Married	302	66.20
	Single	154	33.80
Age	Between 18 and 30 years old	64	14.00
	Between 31 and 40 years old	198	43.40
	Between 41 and 50 years old	167	36.60
	51 years and over	27	6.00
Education status	Bachelor’s degree	381	83.60
	Postgraduate	75	16.40
Experience	5 years and below	48	10.50
	Between 6 and 10 years	103	22.60
	Between 11 and 15 years	158	34.60
	Between 16 and 20 years	92	20.20
	21 years and over	55	12.10
Income	Between 60.000 and 70.000 Turkish Lira	122	26.80
	Between 70.001 and 80.000 Turkish Lira	87	19.00
	Between 80.001 and 90.000 Turkish Lira	92	20.20
	Between 90.001 and 100.000 Turkish Lira	97	21.30
	100.001 Turkish Lira and over	58	12.70

There are some prerequisites for determining the validity and reliability of the findings obtained within the scope of the research. Kurtosis and skewness values between (±) 1.96 indicate that the sample will be normally distributed. Factor loading values greater than 0.50, Cronbach’s Alpha, Composite Reliability (CR), and rho_A coefficients greater than 0.70, and Average Variance Extracted (AVE) value greater than 0.50 are important indicators of validity and reliability. A Variance Inflation Factor (VIF) value below 3 within the scope of the question items indicates that there is no common method bias (Hair et al., 2022). Table 6 presents the related values in detail.

**Table 6:** Factor loading, validity, and reliability values.

Items	Factor loading	Mean	Standard deviation	Kurtosis	Skewness	VIF
Sustainable Leadership Scale Cronbach’s Alpha = 0.869, rho_A = 0.875, CR = 0.902, AVE = 0.607						
SL1	0.780	3.559	1.031	0.202	−0.774	1.824
SL2	0.750	3.638	0.931	0.444	−0.759	1.722
SL3	0.802	3.708	0.934	0.536	−0.812	1.994
SL4	0.812	3.603	0.997	0.064	−0.699	2.080
SL5	0.689	3.384	1.057	−0.534	−0.379	1.488
SL6	0.832	3.524	0.998	0.027	−0.639	2.217
AI Perception Scale Cronbach’s Alpha = 0.859, rho_A = 0.862, CR = 0.890, AVE = 0.503						
AI1	0.646	3.599	1.010	0.068	−0.693	1.381
AI3	0.721	3.480	0.984	−0.314	−0.402	1.764
AI4	0.643	3.493	0.982	−0.167	−0.504	1.565
AI6	0.713	3.583	0.997	−0.060	−0.611	1.692
AI7	0.749	3.496	0.971	−0.092	−0.579	1.755
AI8	0.720	3.614	0.944	0.620	−0.873	1.708
AI9	0.766	3.487	1.002	0.110	−0.685	1.862
AI10	0.706	3.636	1.000	0.323	−0.794	1.566
Digital Transformation Scale Cronbach’s Alpha = 0.862, rho_A = 0.862, CR = 0.906, AVE = 0.708						
DT1	0.808	3.577	1.017	0.063	−0.636	1.797
DT2	0.854	3.522	1.032	−0.020	−0.599	2.148
DT3	0.863	3.522	1.000	0.059	−0.635	2.322
DT4	0.839	3.550	0.994	0.112	−0.644	2.086
Creative Idea Generation Scale Cronbach’s Alpha = 0.806, rho_A = 0.814, CR = 0.873, AVE = 0.633						
CI1	0.797	3.636	0.991	0.373	−0.768	1.646
CI2	0.828	3.594	0.944	0.457	−0.777	1.807
CI3	0.724	3.535	1.028	−0.255	−0.533	1.448
CI4	0.827	3.621	0.961	0.360	−0.725	1.742
Circular Economy Awareness Scale Cronbach’s Alpha = 0.905, rho_A = 0.906, CR = 0.924, AVE = 0.602						
CE1	0.765	3.662	1.066	0.094	−0.766	1.876
CE2	0.794	3.621	1.004	0.030	−0.647	2.204
CE3	0.813	3.673	0.980	0.197	−0.708	2.367
CE4	0.776	3.702	0.993	0.128	−0.670	2.058
CE5	0.747	3.667	0.993	0.154	−0.697	1.795
CE6	0.766	3.546	1.081	−0.029	−0.715	1.937
CE7	0.777	3.693	0.968	0.206	−0.649	1.976
CE8	0.767	3.662	0.991	−0.002	−0.625	1.933

When Table 6 is examined, it is seen that the sample is normally distributed in light of kurtosis and skewness values. Since the factor loadings of AI2 and AI5 were below 0.50, they were removed from the scale. It is seen that all of the scales provide Cronbach’s Alpha, CR, AVE, and rho_A values. Since the VIF value of the statements used in the study was below 3, it was determined that there was no common method bias for the statements.

In order to make sense of the analyses, it is not enough for the validity, reliability, and common method bias to be at the specified values; also, the discriminant validity of the scales should be tested. When the literature is examined, the discriminant validity proposed by Fornell and Larcker (1981) and the HTMT (Heterotrait-Monotrait) ratio are among the most frequently used methods among the criteria (Hair et al., 2022). The discriminant validity values calculated based on the Fornell and Larcker criteria and the HTMT ratio are presented in Table 7.

**Table 7:** Discriminant validity values.

Fornell-Larcker criterion and HTMT ratio					
Items	1	2	3	4	5
AI Perception	0.709	0.686*	0.656*	0.680*	0.686*
Circular Economy Awareness	0.614	0.776	0.831*	0.825*	0.745*
Creative Idea Generation	0.556	0.712	0.795	0.829*	0.832*
Digital Transformation	0.589	0.730	0.695	0.841	0.889*
Sustainable Leadership	0.604	0.665	0.701	0.788	0.779

* HTMT Ratio.

As a result of the test, it was determined that the separation between the scales was at a sufficient level. With this result, the mediation analysis was conducted by selecting a sample size of 5,000 with the Bootstrapping method in the SmartPLS program to test the model (Hair et al., 2022). The bootstrapping method was developed by Efron (1992) and generates 5,000 resamples from the original sample, providing an important foundation for mediation analysis. The model goodness-of-fit values are presented in Table 8.

**Table 8:** Goodness-of-fit values.

Model fit		
Items	Saturated model	Estimated model
SRMR	0.056	0.063
d_ULS	1.446	1.854
d_G	0.550	0.580
Chi-Square	1.398.248	1.428.441
NFI	0.828	0.824

When Table 8 is examined, the SRMR value is below 0.080, and the NFI value is above 0.80, indicating that the model meets the goodness-of-fit criteria (Byrne, 2016). The visualization of the model is presented in Figure 2.

**Figure 2:**
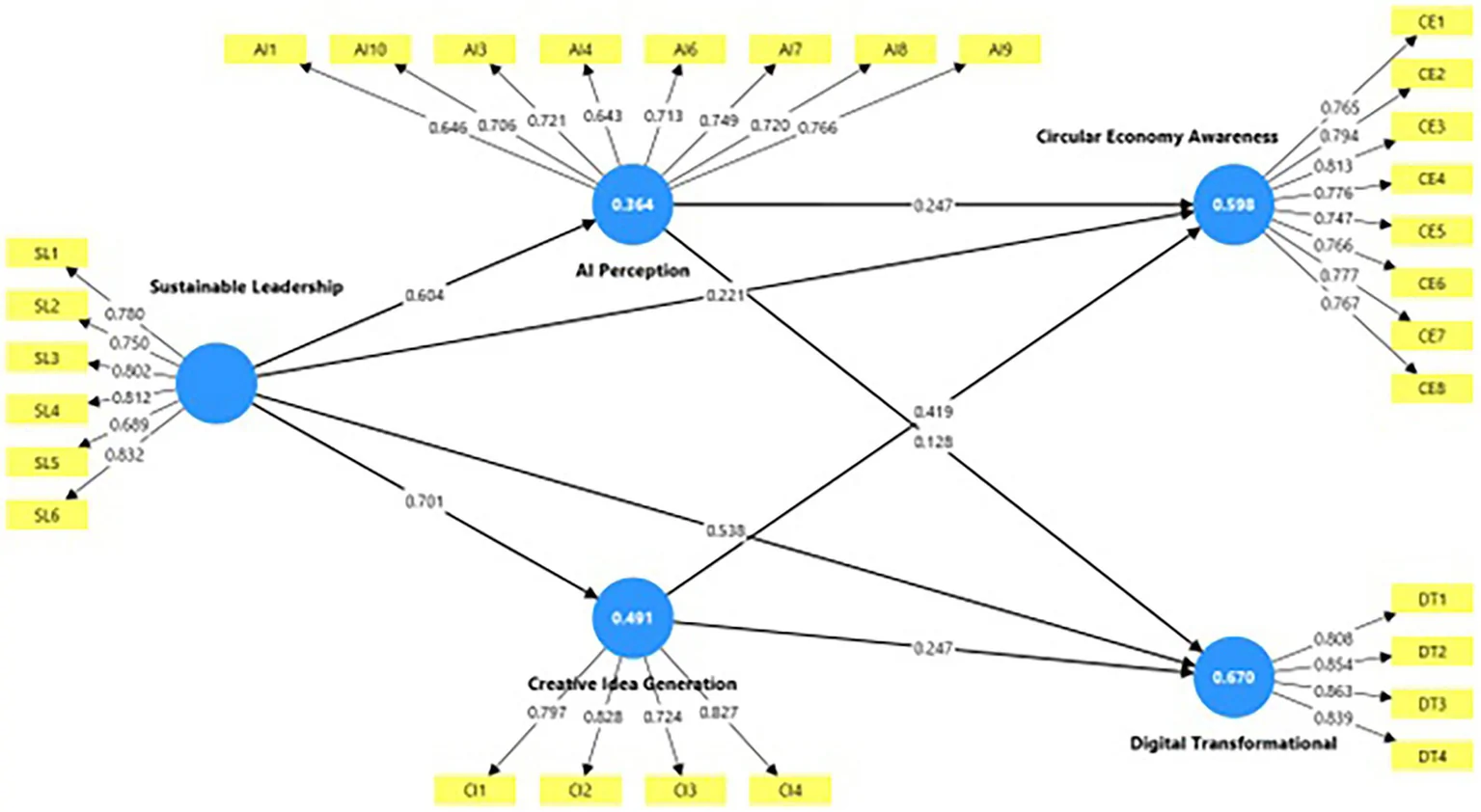
Path Diagram.

Hypothesis tests were conducted when the results obtained were appropriate. The values related to hypothesis tests are presented in Table 9. Regarding the hypothesis tests, t values above 1.96 and *p* values below 0.05 at the 95% confidence level indicate that the hypothesis is confirmed. VAF is used not as a significance criterion for accepting or rejecting mediation, but as an indicator of the magnitude/ratio of the indirect effect. In mediation tests, a Variance Accounted For (VAF) value between 20 and 80% indicates partial mediation, while a value between 80 and 100% indicates full mediation (Hair et al., 2022). The f^2^ value serves as a standardized average effect measure for participants at all levels of the independent variable. An f^2^ value of 0.02 or greater indicates a low-level relationship, 0.15 or greater indicates a medium-level relationship, and 0.35 or greater indicates a high-level relationship (Hair et al., 2022).

**Table 9:** Hypothesis test results.

Path	ß	Standard deviation	*t*-value	*p*	Hypothesis	BC %95 CI	f^2^-VAF
Sustainable Leadership → AI Perception	0.604	0.040	15.185	0.000	H1 Accept	(0.515–0.674)	0.573
Sustainable Leadership → Circular Economy Awareness	0.221	0.068	3.271	0.001	H2 Accept	(0.088–0.350)	0.054
Sustainable Leadership → Digital Transformation	0.538	0.071	7.589	0.000	H3 Accept	(0.384–0.661)	0.388
Sustainable Leadership → Creative Idea Generation	0.701	0.039	17.837	0.000	H4 Accept	(0.610–0.765)	0.966
AI Perception → Circular Economy Awareness	0.247	0.055	4.465	0.000	H5 Accept	(0.138–0.356)	0.091
AI Perception → Digital Transformation	0.128	0.046	2.770	0.006	H6 Accept	(0.037–0.216)	0.030
Creative Idea Generation → Circular Economy Awareness	0.419	0.066	6.396	0.000	H7 Accept	(0.281–0.541)	0.210
Creative Idea Generation → Digital Transformation	0.247	0.072	3.428	0.001	H8 Accept	(0.122–0.405)	0.089
Sustainable Leadership → AI Perception → Circular Economy Awareness	0.149	0.034	4.385	0.000	H9 Accept partial	(0.084–0.217)	VAF: 0.403
Sustainable Leadership → AI Perception → Digital Transformation	0.077	0.030	2.608	0.009	H10 Accept Partial	(0.022–0.137)	VAF: 0.126
Sustainable Leadership → Creative Idea Generation → Circular Economy Awareness	0.294	0.046	6.441	0.000	H11 Accept partial	(0.084–0.295)	VAF: 0.570
Sustainable Leadership → Creative Idea Generation → Digital Transformation	0.173	0.053	3.247	0.001	H12 Accept partial	(0.084–0.217)	VAF: 0.243

When Table 9 is analyzed, hypothesis H1 is accepted since sustainable leadership has a significant effect on AI Perception (ß = 0.604; *p* < 0.05). The f^2^ value of 0.573 between the two variables shows that the effect is at a high level. Since sustainable leadership has a significant effect on circular economy awareness (ß = 0.221; *p* < 0.05), hypothesis H2 is accepted. The f^2^ value between the two variables is 0.054, indicating that the effect is at a low level. Since sustainable leadership has a significant effect on digital transformation (ß = 0.538; *p* < 0.05), hypothesis H3 is accepted. The f^2^ value between the two variables is 0.388, indicating that the effect is at a high level. Since sustainable leadership has a significant effect on creative idea generation (ß = 0.701; *p* < 0.05), hypothesis H4 is accepted. The f^2^ value of 0.966 between the two variables shows that the effect is at a high level. Since there is a significant effect of AI perception on circular economy awareness (ß = 0.247; *p* < 0.05), hypothesis H5 is accepted. The f^2^ value between the two variables is 0.091, indicating that the effect is at a low level. Since there is a significant effect of AI perception on digital transformation (ß = 0.128; *p* < 0.05), hypothesis H6 is accepted. The f^2^ value between the two variables is 0.030, indicating that the effect is at a low level. Since there is a significant effect of creative idea generation on circular economy awareness (ß = 0.419; *p* < 0.05), hypothesis H7 is accepted. The f^2^ value between the two variables is 0.210, indicating that the effect is moderate. Since there is a significant effect of creative idea generation on digital transformation (ß = 0.247; *p* < 0.05), hypothesis H8 is accepted. The f^2^ value between the two variables is 0.089, indicating that the effect is at a low level. The partial mediating effect of AI Perception on the effect of sustainable leadership on circular economic awareness (ß = 0.149; *p* < 0.05) was determined since the VAF value was higher than 20%, and hypothesis H9 was accepted. Since the partial mediating role of perceptions of artificial intelligence regarding the impact of sustainable leadership on digital transformation (ß = 0.077; *p* < 0.05) was statistically significant, Hypothesis H10 was accepted. Statistically, the hypothesis should be accepted; however, some studies in the literature indicate that caution is warranted because the VAF value is below 20%. From this perspective, although the authors accepted the hypothesis, there remains a point open to criticism regarding this matter (Balu and Rathnasabapathy, 2025; Sabol et al., 2026). The partial mediating effect of creative idea generation on the effect of sustainable leadership on circular economic awareness (ß = 0.294; *p* < 0.05) was determined since the VAF value was higher than 20% and hypothesis H11 was accepted. The partial mediating effect of creative idea generation on the effect of sustainable leadership on digital transformation (ß = 0.173; *p* < 0.05) was determined since the VAF value was higher than 20%, and hypothesis H12 was accepted.

The most important evaluation criteria for the PLS-SEM structural model are R^2^ (variance explained), f^2^ (effect size), Q^2^ (predictive fitness), and the size and statistical significance of the structural path coefficients. The R^2^ value indicates the predictive power of the model and is also expressed as the squared correlation between the actual and predicted values of the endogenous variable. In the structural model, Q^2^ values greater than zero for a given reflective endogenous latent variable indicate the predictive fit of the path model for a given dependent construct (Hair et al., 2022). R^2^ and Q^2^ test results are presented in Table 10.

**Table 10:** R^2^ and Q^2^ test results.

Items	R^2^	R^2^ Adj.	Q^2^ Adj.
AI perception	0.364	0.363	0.356
Circular economy awareness	0.598	0.595	0.435
Creative idea generation	0.491	0.490	0.485
Digital transformation	0.670	0.668	0.617

R^2^ values greater than 0.25 in Table 10, together with the Q^2^ value greater than zero, provide a prediction in terms of the appropriateness of the model (Hair et al., 2022). With this prediction, it is determined that the model is appropriate and compatible.

## 6 Discussion

This study was conducted to examine the mediating role of AI perception and creative idea generation in the impact of a sustainable leadership approach on digital transformation and circular economy awareness. Analyses using structural equation modeling revealed that the proposed theoretical model showed a high level of fit, and the hypotheses were significantly supported. These findings suggest that sustainable leadership functions as a strategic element not only in the context of social responsibility and environmental awareness but also in digital transformation processes. From this point of view, it is understood that sustainable leadership paves the way for current practices in addition to leadership that is considered in terms of responsibility and consciousness. It also shows that when organizations are managed with a vision that focuses not only on profit but also on social and environmental values, digital transformation processes progress more healthily.

The main claim of the research is that by assuming a designing role within the scope of Dynamic Capabilities Theory, the organization restructures its artificial intelligence, human creativity, and technological infrastructure resources according to rapidly changing global market conditions (Teece, 2025). In addition, the execution of circular economy processes without systematic and holistic planning may lead to a decline in product and service quality, which may result in negative outcomes such as decreased customer satisfaction and customer loss. Therefore, the successful implementation of circular economy-based transformation is possible not only through technical practices but also through a leadership approach that is shaped by a green transformation perspective and can strategically guide the process (Bashynska et al., 2024).

AI technologies provide significant advantages in terms of speed, efficiency, and data analytics in today’s business world. However, it can be said that it also leads to a transformation in the understanding of leadership. Especially in terms of leadership, it is reported that they should evaluate AI applications not only as an automation tool but also as an innovation within the framework of ethical principles (George et al., 2021). In addition, recent studies suggest that leadership in today’s age of AI should reveal more than a traditional form and should go beyond communication models (He, 2025). Leaders should foster a shared digital culture where hierarchical barriers are minimized, digitalization is at the forefront, and is essential for achieving sustainable competitive advantage (Sagbas et al., 2023). Through their ability to influence organizational culture, allocate resources, and align technological developments with business objectives, some middle and senior managers have a critical role to play in ensuring that AI initiatives are effectively implemented and generate sustainable value (Bevilacqua et al., 2025).

### 6.1 Managerial/practical implications

The results of the structural equation model analysis suggest that employees’ perceptions and orientations towards technology play a critical psychological role in facilitating the impact of leadership approaches on organizational outcomes. In the context of the mediation relationship, sustainable leadership, in addition to creating direct effects, creates an indirect effect by shaping employees’ cognitive and emotional attitudes towards technological systems. In this context, leadership is considered not only as a strategic direction setter but also as an attitude shaper. New and creative ideas are vital in the transformation processes of organizations, especially in the adoption of circular economy practices and the implementation of projects (Frolova et al., 2021). Sustainable leaders can ensure the development and implementation of these new ideas by increasing circular economy awareness and encouraging innovative behaviors among employees (Ahsan and Khawaja, 2024). In the process of implementing innovative behaviors, sustainable leaders adopt it as a fundamental priority that business processes are compatible with sustainability and circular economy principles without harming the social and physical environment (Vrontis et al., 2023). Innovative behavior refers to employees generating creative ideas focused on sustainability and the circular economy, developing these ideas, and transforming them into solutions that can be applied to organizational processes. In this respect, it is usual for the creative idea to play a mediating role.

The partial mediating effect of AI perception on the effect of sustainable leadership on circular economy awareness reflects the ability of sustainable leaders to harmonize technological tools with environmental goals. It is emphasized in the literature that sustainable leaders transform employees’ perspective on technology by adopting a “responsible innovation” approach (Waqar et al., 2023). Sustainable leadership uses AI as a tool that creates resource efficiency and makes systems smart. The positive perception that employees develop towards AI can create a psychological ground that increases awareness of the “zero waste” and “resource cycle” principles of the circular economy (Payer et al., 2024). On the other hand, when the partial mediating effect of creative idea generation on the effect of sustainable leadership on circular economy awareness is examined, it can be explained by the idea that sustainability requires out-of-the-box solutions that eliminate conventional explanations. Sustainable leaders and sustainability understanding create a vision within the organization and encourage employees to question existing production models and design greener alternatives (Geissdoerfer et al., 2020). This process transforms the circular economy from a theoretical concept into a concrete awareness in product design and workflows. It should also be noted that the institutionalization of sustainability requires creativity triggered by leadership.

The partial mediation effect of creative idea generation in the effect of sustainable leadership on digital transformation reveals that creative idea generation also builds a critical bridge in the relationship between sustainable leadership and digital transformation. Although the H10 hypothesis is conceptually and theoretically significant according to the bootstrapping mediation method (β = 0.077, *p* = 0.009, 95% CI [0.022, 0.137]), the statistically low VAF value is a major source of criticism (Sabol et al., 2026). The limited nature of the findings stems from the fact that digital transformation, including the rewriting of organizational DNA, direct leadership intervention, and strategic resource allocation, plays a far more dominant role than employees’ individual perceptions of technology (Warner and Wäger, 2019). Furthermore, given the complexity of artificial intelligence technologies and the pace at which they are integrated into organizational culture, it is evident that awareness at the level of “perception” alone does not constitute a sufficiently effective “transmission mechanism” to transform the organizational impact of leadership (Yu et al., 2025). In light of this finding, it is clear that leaders seeking to contribute to digital transformation and disseminate it throughout the organization must not only manage employees’ attitudes but also adapt sustainable leadership practices to the organization’s infrastructure. Additionally, the use of numerous variables in the statistical analysis is believed to have influenced this result.

Although the findings suggest that sustainable leadership appears similar to the concepts of transformational leadership, responsible leadership, ethical leadership, and green leadership, they indicate that sustainable leadership differs from other leadership concepts in terms of its fundamental objectives, theoretical foundation, and temporal perspective on management. It is evident that sustainable leadership distinguishes itself from other types of leadership by focusing on the concepts of economic, social, and environmental sustainability. At the same time, it is seen to create long-term, multi-stakeholder value through practices and applications aimed at how the organization will create value in the long run. The study’s findings confirm that the specified characteristics of sustainable leadership are indeed present.

### 6.2 Theoretical implications

In terms of theoretical contribution and originality, this study has several contributions. First of all, while the literature on sustainable leadership has mostly focused on environmental performance, corporate social responsibility, and sustainable business outcomes, this study examines sustainable leadership in a holistic model with two critical transformation areas, such as digital transformation and circular economy awareness. Thus, sustainable leadership is not only an ethical and environmental managerial approach, but also a high-level dynamic capability that drives technological and strategic transformation processes. On the other hand, in the existing literature, the relationship between sustainable leadership and digital transformation is mostly analyzed through direct effects. This study addresses the gap in the literature by explaining the mechanisms through which this relationship is realized. Testing the mediating role of AI perception and creative idea generation variables shows that the impact of sustainable leadership on digital and circular transformation occurs through two different cognitive and behavioral channels. Finally, the research contributes to the foundations of the Dynamic Capabilities Theory by positioning sustainable leadership in the context of the capacity to restructure organizational resources, drive technological adaptation, and generate innovation. In terms of the theory, the statistical demonstration of the strong partial mediation effect of creative idea generation shows that dynamic capabilities are shaped through human processes (Teece, 2025).

Digital transformation is a process of “strategic renewal” that inherently requires high levels of innovation and the capacity to deal with uncertainty (Warner and Wäger, 2019). By showing employees the “human and environmental” benefits of this transformation, sustainable leadership channels their creative potential to create digital solutions. In terms of Dynamic Capabilities Theory, creativity represents the most important elements that transform the vision provided by leadership into a digital capability. It can be interpreted that the success of digital transformation depends on the leader’s building a “people-oriented” organizational climate that will use this technology creatively rather than on technological investments (Walter, 2024).

### 6.3 Limitations and recommendations for future studies

As with any research, this study has certain limitations. Being aware of these limitations enables the findings to be interpreted accurately in terms of generalizability and also suggests new avenues for future research. Given that the sample consists of SMEs from three different provinces across Turkey, there is a limitation regarding the generalizability of the findings. The design of this study was quantitative, which is an important limitation in terms of revealing cultural and contextual differences. In order to analyze these differences in more depth, it is recommended to use methods based on qualitative data sources. In addition, this study, which uses a data collection model with a specific time period, is insufficient to draw conclusions about how the relationships between variables change or develop over time. It is predicted that this limitation can be eliminated in studies to be conducted over multiple time periods. As mentioned above, studies between concepts were handled separately, but in this study, multiple variables were brought together. Although many variables are considered in a single model, the literature shows that various factors, such as change management, the technology acceptance model, and ethical concerns, are effective in similar processes. Future studies can consider these different psychosocial variables as mediating or moderating factors, and thus make the model more comprehensive. In addition, the instrument used for data collection may have limitations such as social desirability bias and respondent bias, as the delivery of the instrument to the participants and their responses to this data collection instrument are based on their subjective perceptions. To greatly reduce these limitations, qualitative interviews, observations, or performance appraisal systems that provide objective data are recommended.

In addition, the research offers critical strategic implications for practitioners. Managers need to approach digital transformation not only as a technical infrastructure change but also as a cultural evolution shaped by a vision of sustainable leadership. Building an organizational climate that supports employees’ capacity for creative idea generation, especially in AI integration processes, will facilitate the achievement of complex sustainability goals such as the circular economy. Accordingly, it is recommended that human resources policies be restructured to combine green talent management and digital literacy competencies. It is also recommended that the study in question be expanded to include the DeLone and McLean IS Success Model, Hierarchy of Quality Dimensions Theory, and the Revised Artificial Intelligence Device Adoption (RAIDUA) Model.

## 7 Conclusion

The study has shown that sustainable leadership can directly and indirectly affect both performance levels, technology processes, and creativity by shaping the digital transformation of the organization and employees’ awareness of the circular economy. The findings reveal that the leadership-digital transformation-technology relationships expressed at the theoretical level in the literature are supported at the empirical level, emphasize the new functions of leadership in the digital age, and fill an important gap in the literature by addressing the impact of sustainable leadership on digitalization processes in the context of artificial intelligence. In addition, although the existing literature has generally examined leadership-digital transformation-technology relationships separately, this study evaluates these concepts in a multi-layered and holistic structure. In addition, the model with four different mediating roles offers a theoretical contribution different from the literature to explain the interactions between the concepts.

## Data Availability

The original contributions presented in the study are included in the article/supplementary material, further inquiries can be directed to the corresponding author.
